# Microtubule dynamics in adult retinal ganglion cells and dorsal root ganglion neurons

**DOI:** 10.3389/fnmol.2026.1739387

**Published:** 2026-01-23

**Authors:** Elena Vecino, Menghon Cheah, Jessica C. F. Kwok, Xandra Pereiro, Noelia Ruzafa, Laura Prieto-López, Richard Eva, Keith R. Martin, James W. Fawcett

**Affiliations:** 1 Experimental Ophthalmo-Biology Group, Department of Cell Biology and Histology, University of the Basque Country, UPV/EHU, Leioa, Spain; 2 Begiker-Ophthalmology Research Group, BioBizkaia Health Research Institute, Cruces Hospital, Barakaldo, Spain; 3 Department of Clinical Neurosciences, John van Geest Centre for Brain Repair, University of Cambridge, Cambridge, United Kingdom; 4 Faculty of Biological Sciences, School of Biomedical Sciences, University of Leeds, Leeds, United Kingdom; 5 Institute of Experimental Medicine, Centre for Reconstructive Neuroscience, Czech Academy of Sciences, Prague, Czechia; 6 Wolfson Sensory Pain and Regeneration Centre (SPaRC), Kings College London, London, United Kingdom; 7 Department of Surgery, University of Melbourne, Melbourne, VIC, Australia; 8 Centre for Eye Research Australia, Royal Victorian Eye and Ear Hospital, Melbourne, VIC, Australia

**Keywords:** dorsal ganglion neurons, EB3, microtubules, neuroregeneration, retinal ganglion cells

## Abstract

**Introduction:**

While axon regeneration is very limited in the adult central nervous system (CNS) in vivo, this is not the case in the peripheral nervous system (PNS). Indeed, both CNS and PNS neurons can regenerate in vitro although to varying degrees. Given the role of microtubule stabilization in promoting regeneration, we have examined microtubule polymerization during the regeneration of two types of adult neurons in vitro, retinal ganglion cells (RGCs) from the CNS and dorsal root ganglion (DRG) neurons from the PNS.

**Methods:**

In order to compare microtubule dynamics between these cell types, the density, polymerization rate and orientation of microtubules have been analysed during neurite regeneration in both cell types by analysing GFP-tagged Microtubule End Binding 3 (EB3) protein transfected into the neurons.

**Results:**

The density of EB3 comets and the speed of EB3 movement was similar in both cell types, although only one subtype of RGC regenerated sufficiently long neurites for analysis. In the absence of extracellular substances that could inhibit neurite regeneration, the dynamics of the microtubules of the RGC subtype that extend long neurites are very similar to those in DRG neurons. However, some RGCs with very short neurites exhibited EB3 comets that progressed retrogradely. Additionally, live imaging of mitochondria was performed in both neuronal cultures.

**Discussion:**

Regenerating neurites assessed in our study exhibited similar microtubule extension dynamics in both CNS- and PNS-originated neurons. Importantly, the observation that robust neurite outgrowth is restricted to RGC subtypes highlights the need to integrate molecular heterogeinity among RGCs in future studies.

## 1 Introduction

Neurons of the peripheral nervous system (PNS) have the capacity to regenerate axons following axotomy, in contrast to neurons of the central nervous system (CNS), where axonal regeneration often fails. This failure in the CNS has been attributed to an extrinsic inhibitory environment that contains myelin and extracellular matrix-associated inhibitors (Geoffroy and Zheng, 2014; Kwok et al., 2014), peripheral macrophages, which have been shown to modulate microglial activity, reducing inflammation after CNS injury (Greenhalgh et al., 2018),in addition to intrinsic limitations in neuronal growth capacity (Mahar and Cavalli, 2018). Therefore, the differential ability of CNS and PNS axons to regenerate depends on interactions with the environment and their subsequent intracellular responses (Varadarajan et al., 2022). Retinal ganglion cells (RGCs), which are part of the CNS, exhibit limited regenerative capacity both *in vitro* (Bahr et al., 1988) and *in vivo*, where numerous strategies have been explored to enhance their regeneration, including treatments that suppress microglial activation (Thanos et al., 1993) and lens injury, which has been found to promote axonal growth (Leon et al., 2000; Yin et al., 2019). Despite these efforts, the number of RGCs capable of regenerating axons remains limited, even when both extrinsic and intrinsic pathways are targeted (Jacobi et al., 2022; Wong and Benowitz, 2022).

Adult RGC axons are capable of regenerating *in vitro* from explants (Bahr et al., 1988; Bahr et al., 1989) and from dissociated cells (Garcia et al., 2002; Garcia and Vecino, 2003; Vecino et al., 2015; Luo et al., 2001). However, not all RGCs exhibit the same regenerative capacity, either *in vivo* (Bahr et al., 1992) or *in vitro* (Garcia et al., 2003; Vecino et al., 2015). Recent studies have demonstrated that silencing 40 specific genes can enhance axonal regeneration after optic nerve crush, though this effect is limited to a subset of RGCs, and they have also identified distinct genetic programs associated with regeneration and cell survival (Lindborg et al., 2021; Jacobi et al., 2022; Tian et al., 2022). RGCs have been classified into at least 46 distinct cell types (Goetz et al., 2022) based on physiological, morphological, and molecular characteristics (Sanes and Masland, 2015); this number increases further when transcriptomic profiles are taken into account (Rheaume et al., 2018).

Microtubules (MTs) are key regulators of axon regrowth (Murillo and Mendes Sousa, 2018) and their stability and dynamics differ markedly between the CNS and the PNS (Ruschel and Bradke, 2018; Zhang and Rasband, 2016; van de Willige et al., 2016; Gobrecht et al., 2016; Kapitein and Hoogenraad, 2015). Following peripheral nerve injury, there is a rapid increase in the number of dynamic MTs within 24 h. In contrast, CNS injury leads to a reduction in tubulin acetylation—an indicator of stable MTs—accompanied by an increase in tubulin tyrosination, which is associated with more dynamic, less stable MTs (Cho and Cavalli, 2012). Neurons also display a distinct polarized organization of MTs: in dendrites, MTs are arranged with dynamic plus ends oriented both toward the cell body and distally, whereas in axons they are uniformly oriented with all plus ends directed toward the growth cone (Baas et al., 1988; Baas and Lin, 2010). The dynamic behavior of microtubules is tightly regulated by numerous intracellular factors, including microtubule-associated proteins (MAPs) Among them, end-binding protein 3 (EB3) is a key MAP that selectively associates with the growing plus ends of microtubules, serving as a marker of dynamic MTs and playing a crucial role in modulating their stability and interactions (Chuang et al., 2014).

The role of MTs in neurite regeneration within the adult CNS *in vivo* remains poorly understood, largely due to the limited regenerative capacity of adult CNS neurons. Most *in vitro* studies on CNS regeneration have utilized embryonic neurons, such as hippocampal, cortical, and Purkinje cells (Petrova et al., 2020; Nieuwenhuis et al., 2020; Koseki et al., 2017; Ferreira and Caceres, 1989; Eva et al., 2017). Conversely, extensive research has been conducted on neurons from the adult PNS, particularly DRG neurons, which retain robust regenerative potential both *in vivo* and *in vitro* (Palmisano et al., 2019). In this study, we compared MT polymerization dynamics in adult rat RGCs and adult rat DRG neurons using *in vitro* assays. We focused on the subset of RGCs capable of extending long neurites during the first 4 days in culture (Vecino et al., 2015). Specifically, we quantified and compared the number, velocity, and proportion of polymerizing MTs in RGCs and DRG neurons. To assess MT dynamics, we employed EB3-GFP, a fluorescent marker that selectively binds to the growing plus ends of MTs, enabling visualization and quantification of MT polymerization events (Kleele et al., 2014). This approach allowed us to measure MT dynamics in both axons and dendrites at various distances from the soma, thereby enabling spatial comparisons of regenerative activity at proximal, distal, and somatic regions from neurons from the CNS and PNS.

## 2 Materials and methods

### 2.1 Animals

All animal experiments adhered to the ARVO Statement for the Use of Animals in Ophthalmic and Vision Research, and to the United Kingdom Animals (Scientific Procedures) Act of 1986. All methods were approved by the University of Cambridge Animal Ethics Committee and conform to the “Guiding Principles for research Involving Animals and Human” as adopted by the American Physiological Society. Adult female Sprague–Dawley rats (250 g–300 g, Harlan Laboratories, Inc.) were used in the present study, and they were housed on a 12-h light–dark cycle with *ad libitum* access to food and water. Animals were sacrificed humanely by exposure to CO_2_. For a 5-liter chamber, a flow rate corresponding to 50% CO₂ was applied. Both RGCs and DRG cultures were established from the same animal. Once the RGCs were in culture, the DRG culture was set-up, only a few hours post-mortem. All experiments presented in the study were repeated at least 4 times.

### 2.2 Retinal ganglion cell culture

The eyes from the adult Sprague Dawley rats were removed, their retinas dissected out and dissociated using the papain dissociation kit (Worthington, Lakewood, NJ, United States). Briefly, the retinas were incubated for 90 min at 37 °C with papain and DNase (1:10), and the cells were dissociated and the resulting cell suspension was transferred to a new tube for centrifugation at 300 x g for 5 min at room temperature. The supernatant was then discarded, and the pellet was resuspended in a solution containing EBSS, albumin ovomucoid inhibitor and DNase. The cell suspension was centrifuged for 6 min. at 70 x g through a discontinuous density gradient prepared with albumin ovomucoid inhibitor at room temperature. The pellet collected was subsequently re-suspended in Neurobasal A supplemented with B27 and L-glutamine (2 mM) to transfect the cells with the expression constructs encoding EB3. After transfection the cells were plated onto poly-L-lysine (PLL, 20 μg/mL) and laminin (10 μg/mL) (Sigma-Aldrich, St. Louis, MO, USA) coated plates at a density of 100,000 cells/well (in 24 well plate), and cultured in Neurobasal A medium supplemented with B27, 2 mM L-glutamine and gentamicin (1:200) (Life Technologies, Carlsbad, CA, USA). The RGCs were maintained at 37 °C in 5% CO_2_ for 4 days until the EB3 recording was obtained.

### 2.3 Dorsal root ganglia culture

DRG were dissected from the adult Sprague Dawley rats, harvested along the entire spinal cord. The DRGs were then incubated for 90 min in 0.2% type IV collagenase (Sigma-Aldrich, St. Louis, MO, USA) in HBSS medium (Life Technologies, Carlsbad, CA, USA) at 37 °C and then with 0.1% trypsin (Sigma-Aldrich, St. Louis, MO, USA) added for a further 10 min. The DRG cells were dissociated using Pasteur pipettes of decreasing diameter and the resulting cell suspension was then centrifuged at 2000 rpm for 2 min. The cell pellet was collected, resuspended, and centrifuged through a 15% BSA (Sigma-Aldrich, St. Louis, MO, USA) density gradient at 1000 rpm for 15 min. Subsequently, the cell pellet was further resuspended and centrifuged at 2000 rpm for 2 min before the cells were plated or transfected by Neon Electroporator (Thermo Fisher Carlsbad, CA, USA). For transfection, the dissociated neurons were re-suspended in 2 mL of Mg^2+^/Ca^2+^-free PBS (phosphate buffered saline, pH 7.0) before they were transfected with expression constructs encoding EB3. The cells were cultured overnight in serum/antibiotic free DMEM medium (Life Technologies, Carlsbad, CA, USA) with poly-L-lysine (20 μg/mL) and laminin (10 μg/mL) (Sigma-Aldrich, St. Louis, MO, USA). The medium was replaced next day with the normal culture medium DMEM supplemented with 10% FBS (Fetal bovine serum, Life Technologies, Carlsbad, CA, USA), 1% penicillin–streptomycin-fungizone, and 10 ng/mL NGF (Nerve growth factor, Sigma-Aldrich, St. Louis, MO, USA). The cells were maintained at 37 °C in 5% CO_2_ from 1 to 3 days in culture until the EB3 recordings were obtained.

### 2.4 Immunocytochemistry

After recording the videos of the EB3 from the neurons dissected from the retina we did immunocytochemistry with antibodies directed to βIII-tubulin in order to verify that the cells transfected and recorded were specifically RGCs, even though the method used to culture RGCs specifically isolate this type of neurons and not the rest of cell types of the retina (Vecino et al., 2015).

After recording, the RGCs neurons were washed twice with PBS and fixed for 10 min with methanol at −20 °C and then washed 3 times with PBS. After blocking the binding of non-specific antigens with blocking buffer (3% BSA and 0.1% Triton X-100 in PBS), the cells were incubated with a rabbit antibody against βIII-tubulin (diluted 1:2,000; Promega, Madison, WI, USA). After washing again, this antibody was detected with an goat anti-rabbit Alexa Fluor 555 secondary antibody (Life Technologies, Carlsbad, CA, USA) diluted 1:1,000, and the cells were counterstained with the nuclear marker DAPI diluted 1:10,000 (Life Technologies, Carlsbad, CA, USA). After washing, the cells were preserved with fluoromount.

### 2.5 DNA expression constructs

The EB3-GFP plasmids constructs have been designed to be expressed in mammals (Akhmanova et al., 2001; Hoogenraad et al., 2000; Perez et al., 1999). To clone the EB3 cDNA, gene-specific primers were designed with in-frame restriction sites for cloning into pEGFP-N1 (Clontech, Palo Alto, CA, USA) after PCR amplification. The primer sequences were based on the human EB3 cDNA, accession numbers: AA2892 (Image clone 714,028). In Western blots of COS-1 cells transfected with the cDNA all the EB3-GFP fusion protein was produced at the expected molecular weight (Hoogenraad et al., 2000; Stepanova et al., 2003).

### 2.6 RGC and DRG neuron transfection

Dissociated RGCs and DRG neurons were transfected using the Electroporator (Life Technologies, Carlsbad, CA, USA), to electroporate cells within a micropipette tip using Neon Transfection Kit (Life Technologies, Carlsbad, CA, USA; MPK1096), applying two 1,200 V pulses of 20 ms duration. Before transfection, the cells were washed briefly in Mg^2+^/Ca^2+^-free PBS and resuspended in the Buffer R provided. For optimum transfection efficiency, 1.0–1.5×10^5^ cells were electroporated with 1 μL of plasmid (500 ng/μl) in 10 μL of transfection buffer. The transfected cells were plated and cultured overnight as described above, without antibiotics or serum, but supplemented with 1% ITS+ (Biosciences, Becton Drive Franklin Lakes, NJ, USA), and it was replaced by normal culture medium the day after transfection.

Four transfections were performed on four different rats, and from the same rat, both RGCs and DRG neurons were transfected on the same day, thereby avoiding possible differences. One and 2 days post transfection (dpt) videos of EB3 were recorded of the DRG and RGCs cultures. RGC cultures were also recorded 4 days post transfection to allow the RGCs to elongate their neurites.

**Figure 1:**
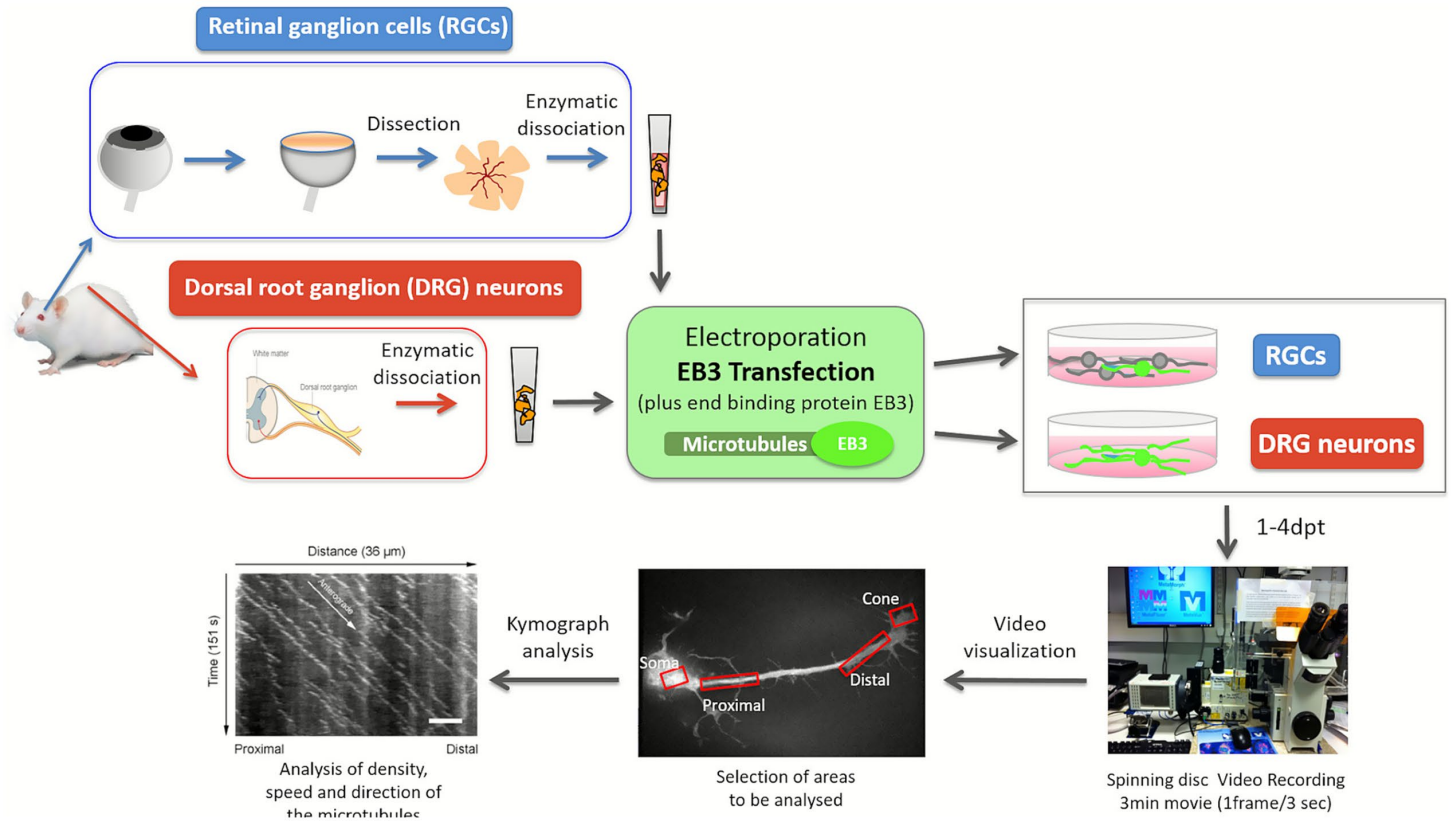
Flow chart of the experimental workflow for analysing microtubule dynamics in retinal ganglion cells (RGCs) and dorsal root ganglion (DRG) neurons. Microtubule activity was assessed by measuring comet density, polymerization speed, and directionality from time-lapse recordings taken 1–4 days post-transfection (dpt). Kymographs were generated from selected regions within the RGCs and DRG neurons. These were analyzed using MetaMorph software to quantify microtubule density, growth speed, and directional behavior.

In RGCs and DRG cultures where the transfection was effective, mitochondria were also labelled with mitoTracker (Life Technologies, Carlsbad, CA, USA; M22426), to test the active transport of organelles after EB3 transfection. The number, speed and direction of the mitochondria was measured from the Kymographs as well as the movement of the EB3 fluorescence that also named comets do to the fluorescence appearance (Figure 1). Mitochondrial transport was used as sign of healthy conditions and was not analysed further.

### 2.7 Live cell imaging, image analysis, and processing

The live imaging of EB3-GFP in adult DRG neurons and RGCs was performed with an Olympus IX70 microscope (Shinjuku, Tokyo, Japan) an UPlan Apo objective 100x/1.35 oil iris and using a Hamamatsu ORCA-ER CCD camera and a PerkinElmer UltraVIEW scanner for spinning disk confocal microscopy, controlled with MetaMorph software. Kymographs were generated using MetaMorph software (Figure 1).

To avoid perturbation of microtubule dynamics attributable to elevated expression of +TIP (plus-end tracking proteins), only neurons exhibiting low EB3–GFP expression were selected for imaging. Low expression was defined by the presence of discrete comet-like labeling restricted to microtubule plus ends, without detectable microtubule-shaft fluorescence or diffuse cytoplasmic signal. Cells showing higher expression were excluded from analysis.

Recordings were taken for 3 min, one frame per every 3 s, with a 491BP filter and the images were analysed by constructing kymographs of each neurite using MetaMorph software (version 7; Molecular Devices). The total width of each neurite was selected for analysis: (a) proximal (10 to 15 μm from the cell body); (b) distal (10 to 15 μm from the growth cone); (c) and in the cell body.

The following parameters of dynamic MTs were measured: density (average number of moving fluorescent comets per μm^2^), speed (average number of moving of EB3 comets by taking in consideration the total displacement divided by time of observation) and directionality (anterograde or retrograde) depending on the angle formed by the comet on the kymograph measured from the orientation set to 0°. To set the directionality of the microtubule polymerization (comet) or the mitochondria, the selected area to be analysed was always proximal to the cell body, thus, all objects that move from left to right, were considered anterograde while those moving from right to left were considered retrograde. Where the angle was more acute in relation to its angle with the horizontal, it meant faster movement, however, if the line described by the object was vertical, it meant no movement. These conditions were considered valid for the MTs as well as for the mitochondria.

### 2.8 MitoTracker staining

The distribution and dynamics of mitochondria in relation to the microtubules in RGC and DRG cells were used exclusively as a control of the stage of health of the cells. For the live mitochondria staining, the cells were incubated with MitoTracker redCMXRos (Life Technologies, Carlsbad, CA, USA) and prepared according to product information. Stock solution was used immediately after it was prepared and dissolved in DMSO to a final concentration of 1 mM. The working concentration was 50 nM in cultured media. For the mitochondrial staining, the culture medium in which the cells were growing after transfection was removed. The cells were incubated for 15 min in the pre-warmed (37 °C) MitoTracker probe and washed with cultured media, live imaging was performed as previously described. Simultaneous video recording for MitoTracker and EB3 were performed with the spinning disk confocal as described above. We performed a statistical analysis of the total number of mitochondria per field in both RGCs (*n* = 118) and DRGs (*n* = 684), classifying them in relation to their motility as stationary, oscillatory or fast. For statistical analysis, mitochondria were normalized to total number per field. This analysis demonstrated the presence of all three types of movement and the overall healthy state of the cells studied.

### 2.9 Statistical analysis

At least three replicates were analysed for each experimental condition, from four independent experiments. Data are expressed as mean values ± standard error of the mean. The sample sizes in the text are given as *n* = neurites analysed, unless stated otherwise. For the values of RGCs and DRG neurons densities, *n* = 20 was analysed for each cell type, and for velocities, *n* = 127 was analysed for DRG neurons and *n* = 150 for RGCs. To assess whether there were significant differences in the EB3 density and transport between the groups, two tailed Student *t*-tests, ANOVA and Bonferroni tests were used. The homogeneity of the variances was assayed with Levene’s test (*p* < 0.05). The minimum value of significance for all tests was defined as *p* < 0.05. All statistical analyses were carried out using IBM SPSS Statistics software v. 21.0 and Microsoft Excel.

## 3 Results

To investigate microtubule dynamics in adult RGCs and DRGs, neurons were transduced with EB3 tagged with GFP and subsequently cultured *in vitro*. The growing “plus-end” tips of polymerizing microtubules appeared as dynamic, fluorescent, comet-like structures as a result of EB3-GFP expression. Using a spinning disk microscope, it was possible to quantify the density of these comets and assess local microtubule dynamics across different neuronal compartments.

**Figure 2:**
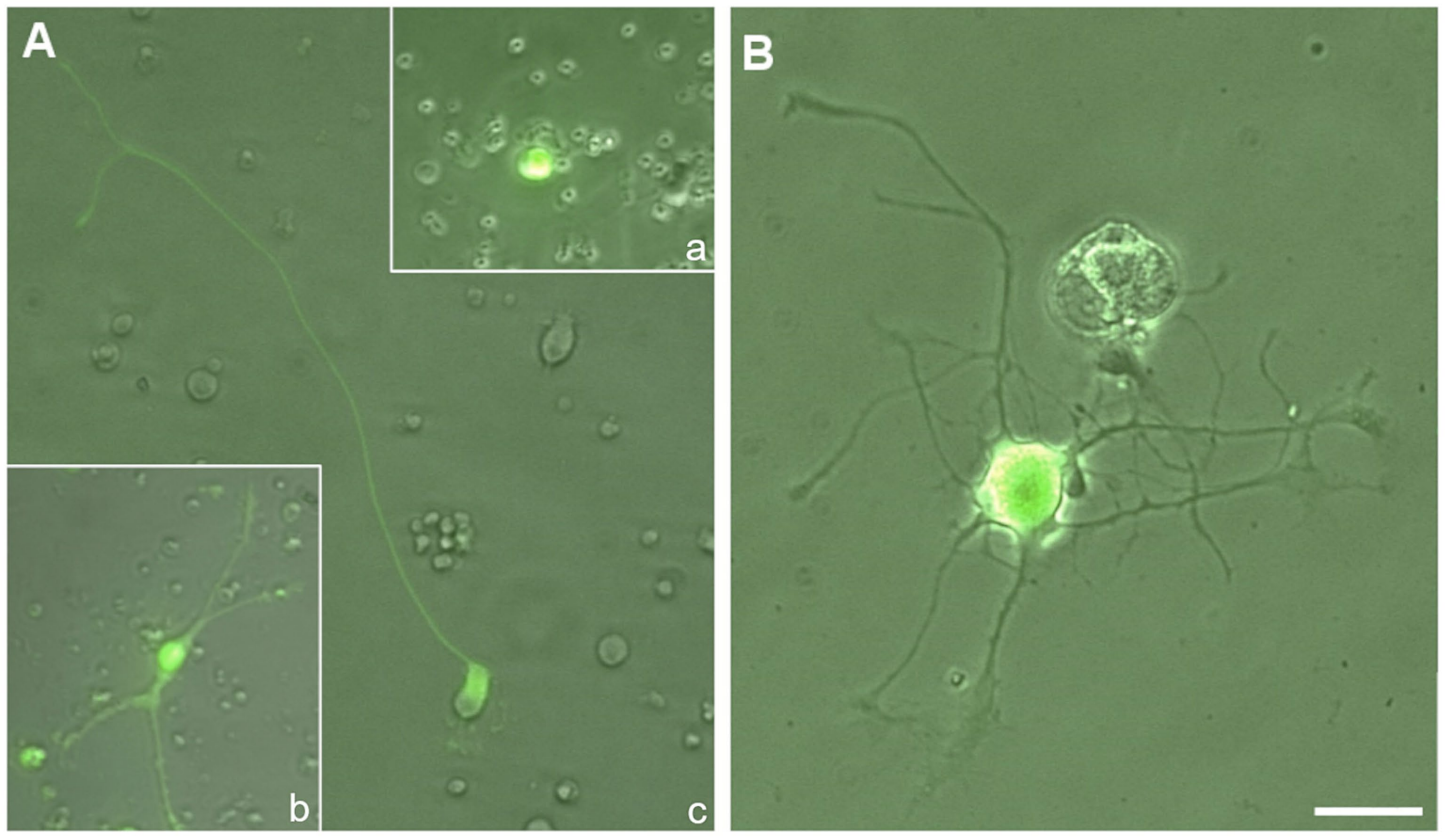
Fluorescence microscopy images of retinal ganglion cells (RGCs) **(A)** and dorsal root ganglion (DRG) neurons **(B)** transfected with EB3-GFP, acquired at 4 and 1 days post-transfection (dpt), respectively. Note the large number and extension of the neurites from DRG neuron compared to that of RGCs. **(A)** RGCs were categorized into three groups based on neurite outgrowth: (a) upper right inset—RGC lacking neurites; (b) lower left inset—RGC with short neurites; and (c) center—primarily monopolar RGCs extending at least one long neurite. Scale bar: 40 μm.

Most RGCs in culture failed to adhere and subsequently died; however, a small subset managed to adhere to the substrate and extend axon-like processes. These surviving neurons typically exhibited a small soma with several neurites, one of which often elongated up to approximately 100 μm and was identified as the axon. Based on neurite morphology, RGCs were categorized into three types: RGCs without neurites, RGCs with short neurites, and predominantly monopolar RGCs that extended at least one long neurite (Figure 2A); the latter group was primarily analyzed in the present study. In contrast, DRG neurons readily extended multiple neurites, all of which were considered axons (Figure 2B). Notably, the number and length of neurites in DRG neurons were significantly greater compared to those in RGCs. In RGCs, neurite emergence was generally delayed, typically occurring around 3 to 4 dpt. By 4 dpt, only a small proportion of RGCs exhibited long neurites; however, in the case of DRG neurons, due to their faster neurite outgrowth, EB3 expression became oversaturated by day 4, and therefore, neurites were analyzed within the first 3 days post-dissection.

**Figure 3:**
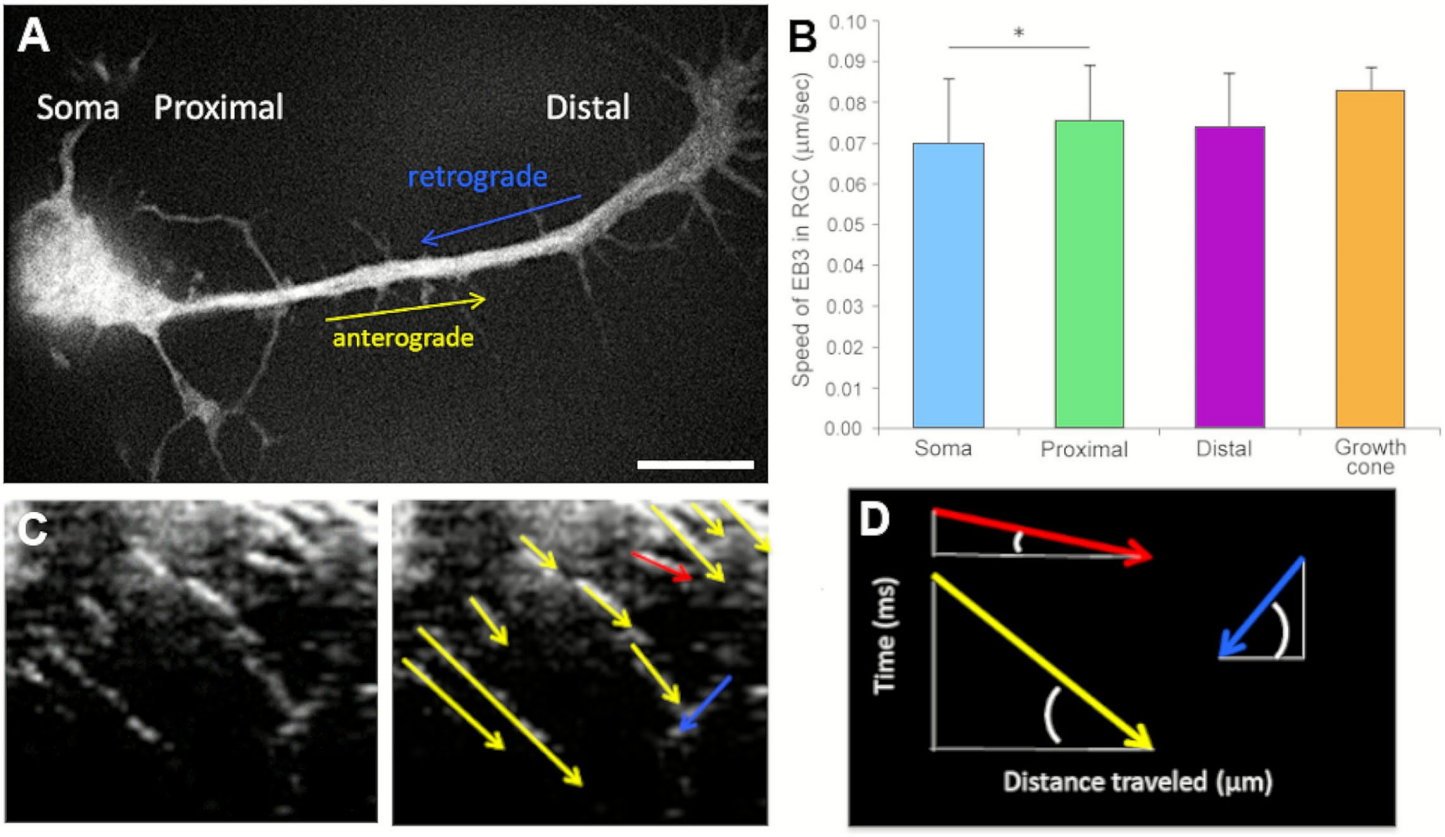
**(A)** Representative snapshot from a time-lapse video of an EB3-GFP–transfected RGC. **(B)** Comet velocities in different cellular regions of RGCs are shown in μm/s. **(C)** Left: kymograph illustrating EB3-GFP comet trajectories; right: quantification derived from the kymograph. **(D)** Diagram showing how the analysis software calculated comet velocity; red and yellow arrows show faster and slower anterograde movements, respectively, while blue arrow represents the retrograde movements of the microtubules. Scale bar: 20 μm, * *p* < 0.05.

The density of EB3-GFP-labeled microtubules was quantified from kymographs generated from time-lapse recordings. Comets within neurites were counted over a standardized area of approximately 30 μm^2^ for both RGCs and DRG neurons. In addition, the directionality and polymerization speed of microtubule growth were analyzed using kymographs of EB3-GFP-labeled neurites. Using MetaMorph software, the distance travelled by individual comets was measured over 3-s recording intervals. Comets moving forward toward the growth cone—representing microtubule plus-ends—were classified as anterograde, whereas those moving in the opposite direction, toward the soma, were defined as retrograde microtubules (Figures 3C,D). Among the three RGC subtypes, only those exhibiting one prominent neurite along with several shorter processes emerging from the soma were selected for analysis. In these cells, we assessed microtubule polymerization dynamics by tracking EB3-GFP comet movement along the neurites, from proximal to distal regions. The speed of comet movement was quantified in four distinct cellular compartments: proximal neurite, distal neurite, growth cone, and soma (Figure 3A). The average polymerization speeds were as follows: proximal neurite, 0.08 ± 0.02 μm/s; distal neurite, 0.08 ± 0.02 μm/s; growth cone, 0.08 ± 0.01 μm/s; and soma, 0.07 ± 0.02 μm/s. Notably, the polymerization speed in the soma was significantly lower than that observed in the proximal neurite (Figure 3B).

**Figure 4:**
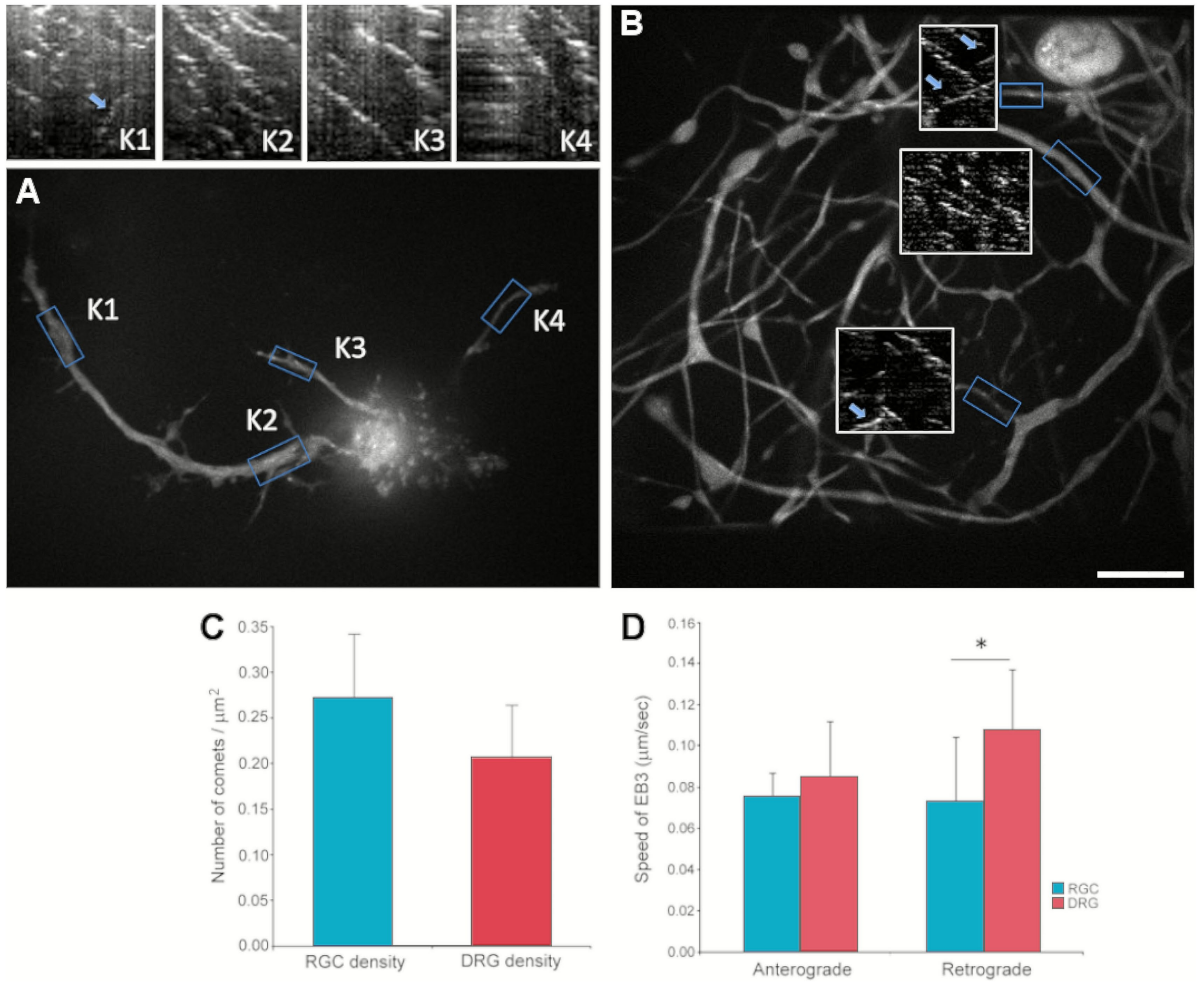
**(A)** Time-lapse image of a transfected RGC recorded at 4 dpt. Rectangular regions indicate analyzed segments; corresponding kymographs are displayed above. The most distal region (K1) shows one retrograde comet (blue arrow). No retrograde comets were observed in more proximal regions (K2–K4) (see Supplementary Video 1). **(B)** Snapshot from a DRG neuron recorded at 1 dpt. Blue boxes mark analyzed neurite regions; white boxes indicate corresponding kymographs. Rare retrograde EB3-GFP comets are marked with blue arrows (see Supplementary Video 2). **(C)** Quantification of comet density, expressed as number of comets per μm^2^ in RGCs and DRG neurons. **(D)** Comet velocity (μm/s) comparison between RGCs and DRG neurons. Scale bar: 40 μm, *p < 0.05.

Furthermore, the density and polymerization speed of EB3-GFP comets were compared between RGCs (Figure 4A, Supplementary Video 1) and DRG neurons (Figure 4B, Supplementary Video 2). Due to the greater length and complex overlapping trajectories of DRG neurites, imaging was performed either near the soma or at distal sites where individual axons were clearly distinguishable. The mean comet density was comparable between RGCs (0.27 ± 0.08 μm^−2^) and DRG neurons (0.21 ± 0.07 μm^−2^) (Figure 4C). Similarly, the average speed of anterograde EB3-GFP-labeled microtubules did not differ significantly between the two cell types: 0.08 ± 0.02 μm/s in proximal RGC neurites and 0.09 ± 0.03 μm/s in DRG neurites. In contrast, retrograde comet speed was significantly higher in DRG neurons (0.11 ± 0.03 μm/s) compared to RGCs (0.07 ± 0.03 μm/s) (Figure 4D). Analysis of comet directionality revealed that 98% of microtubules exhibited anterograde growth in both RGCs (subtype with long neurites) and DRG neurons.

**Figure 5:**
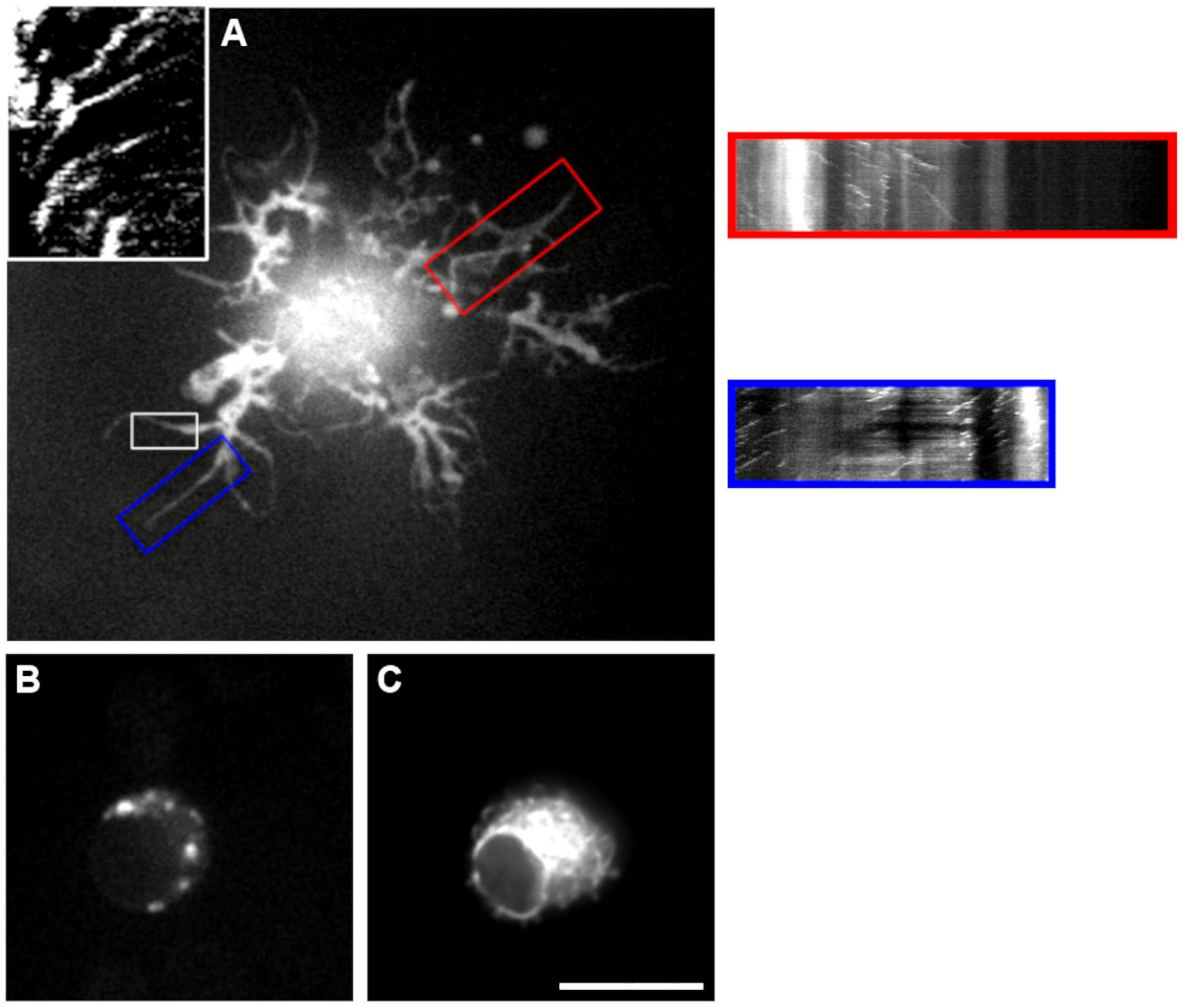
Representative snapshots of transfected small retinal ganglion cells (RGCs) that remained viable but failed to regenerate neurites. **(A)** RGC displaying short neurite outgrowth at 4 days post-transfection (dpt). Three neurites were analyzed, corresponding to regions K1, K2, and K3, and their respective kymographs are shown in white (K1), red (K2), and blue (K3) frames. In K1 and K3, comet trajectories are oriented from right to left, indicating retrograde microtubule movement. In contrast, K2 exhibits a few comets moving anterogradely (left to right), as well as several stationary comets (vertical traces), suggesting reduced microtubule dynamics (see Supplementary Video 3). **(B,C)** Images of the same RGC that did not extend neurites. **(B)** The cell labeled with MitoTracker shows bright, mobile mitochondrial puncta, indicating cellular viability. **(C)** EB3-GFP–transfected image of the same cell reveals dynamic microtubule activity despite the absence of neurite extension. Scale Bar 10 μm.

Besides the RGCs that emit long neurites and where it was possible to measure the density and speed of the microtubules, a second subtype of RGCs transfected developed several short, thin-caliber neurites with secondary branches, typically accompanied by one longer process. In these cells, microtubule dynamics varied by neurite type: comets in the small-caliber neurites predominantly moved in the retrograde direction, whereas those in the larger neurite displayed mostly anterograde growth (Figure 5A, Supplementary Video 3). A third RGC subtype, characterized by a small, rounded soma, failed to elaborate processes. Nevertheless, these cells exhibited abundant mitochondria that moved actively within the cytoplasm (Figures 5B,C).

**Figure 6:**
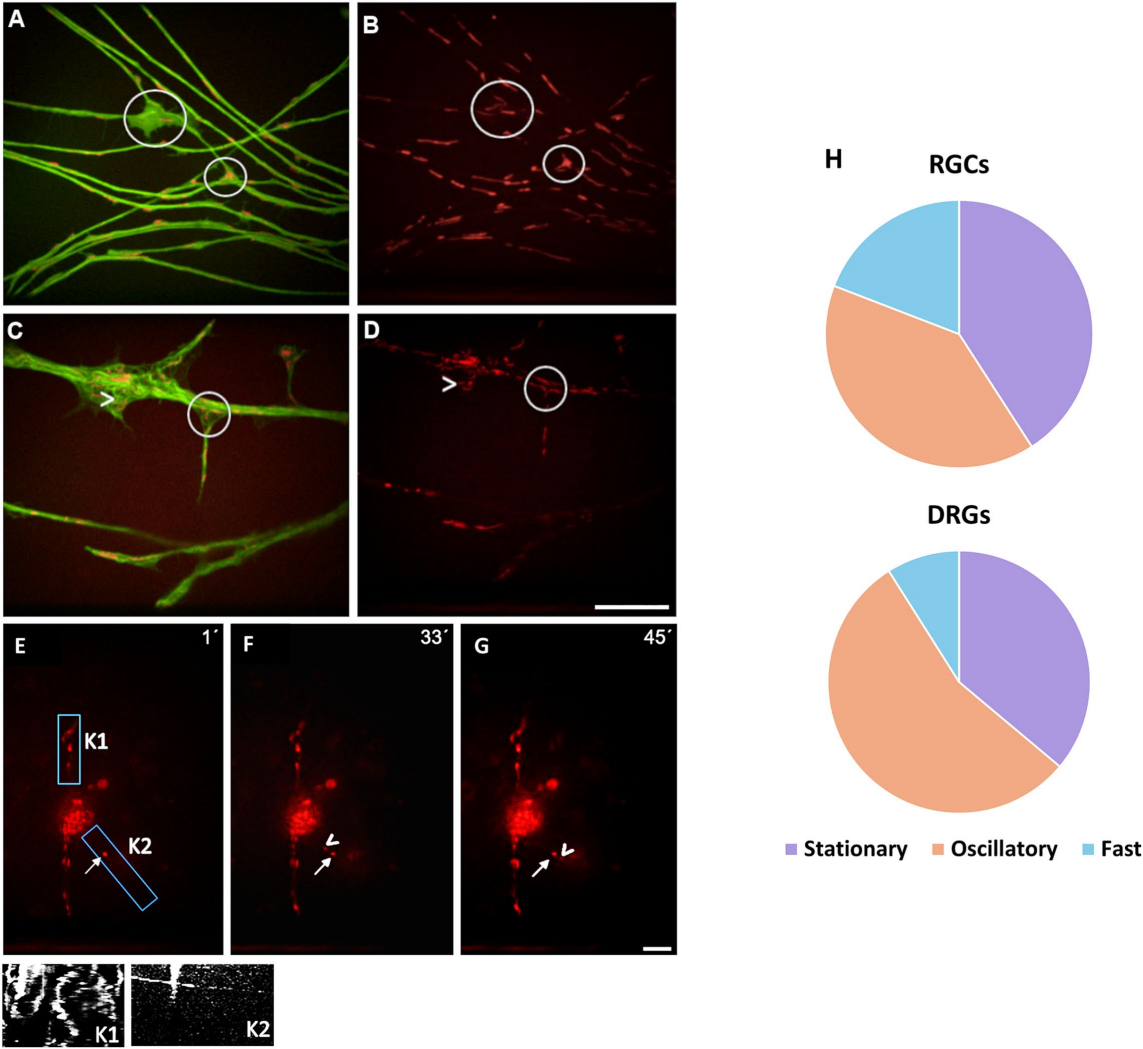
Co-localization of EB3-GFP (**A**, Supplementary Video 4; **C**, Supplementary Video 6) and mitochondria (**B**, Supplementary Video 5; **D**, Supplementary Video 7) in DRG neurons. Numerous mitochondria were observed within neurite branches (circled areas), and in some cases, they conformed to the curved structure of the underlying microtubules (arrows in **C** and **D**). **(E–G)** Time-lapse imaging of mitochondrial movement in a transfected RGC at 1 **(E)**, 33 **(F)**, and 45 **(G)** seconds. Kymographs correspond to the regions marked by rectangles in panel E. In K1, most mitochondria remain stationary or display minimal movement. In contrast, K2 reveals rapid anterograde transport (horizontal line) alongside stationary mitochondria (vertical line) within the same neurite. Some neurites contained a higher density of mitochondria, which generally exhibited reduced motility, as illustrated in K1 (see Supplementary Video 8). **(H)** Quantification of mitochondrial motility (stationary, oscillatory or fast movement) in RGCs and DRGs neurites, respectively, normalized as the percentage of total mitochondria per field. In RGCs 40.85 **±** 9.68% were stationary, 39.48 **±** 11.94% oscillatory and 19.14 **±** 4.36% fast; meanwhile, in DGRs 36.11 **±** 5.93% were stationary, 54.91 **±** 6.14% oscillatory and 8.98 **±** 0.51% fast Scale bars: 10 μm **(A–D)**, 20 μm **(E–G)**.

In addition to EB3-GFP tracking, mitochondrial movement was recorded using the MitoTracker dye. The position and orientation of mitochondria did not correlate always spatially with microtubule polymerization patterns. While the directionality and ratio of microtubule polymerization remained consistent, mitochondrial distribution and motility were highly variable. Mitochondria in DRG neurons appeared longer than those observed in RGCs. Generally, smaller mitochondria exhibited faster movement, predominantly in the anterograde direction. This is in agreement with the higher percentage of fast movement mitochondria observed in RGCs (Figure 6H). Nonetheless, in both neuron types, the largest proportion of mitochondria were either stationary or showed only minimal (oscillatory) displacement. Overall, mitochondrial movement was considered as a proxy for the general dynamics of organelle transport. Representative examples of mitochondrial motility in relation to microtubule polymerization are provided in Supplementary Videos 4–8 (Figure 6).

## 4 Discussion

Unlike PNS neurons, CNS neurons lose their intrinsic regenerative capacity as they mature (Kang and Lichtman, 2013; Varadarajan et al., 2022). However, a subset of adult RGCs can survive in culture and extend long neurites (Vecino et al., 2015). While some mechanisms promote axon regeneration in both the PNS and CNS, such as Akt signaling -which enhances axon regeneration in the PNS and can even induce optic nerve regeneration in the CNS (Guo et al., 2016) -others show system-specific effects. For example, guanine promotes axon growth in DRG neurons *in vitro* and enhances axon regeneration *in vivo*, but does not induce optic nerve regeneration; instead, it improves RGC survival after optic nerve crush injury (Li et al., 2025). In this study, we enabled direct comparisons of microtubule and mitochondrial dynamics in regenerating axons of adult DRG and RGC neurons derived from the same animal.

### 4.1 Diversity of cell types

To visualize EB3 comets during neurite growth, we recorded time-lapse videos of DRG neurons at 1, 2, and 3 days post-transfection (dpt). In contrast, for RGCs, neurite outgrowth was not observed until 4 dpt, with some cells requiring 6 to 7 days *in vitro* to extend long neurites (Garcia et al., 2002; Garcia and Vecino, 2003; Vecino et al., 2015). Only a small fraction of RGCs in culture, approximately 3% of the surviving population, are capable of elaborating long neurites. This rare subset of RGCs may possess distinct regenerative mechanisms and could correspond to the same cells that exhibit long-distance axon growth in the optic nerve following injury, particularly under regenerative treatments (Wang et al., 2012). Furthermore, Dupraz et al. also identified a small population (2–3%) of RGCs capable of extending long neurites after just 3 days *in vitro*, even when IGF-1R (Insulin-like growth factor 1 Receptor) signalling was blocked, a condition that inhibits axonal outgrowth in the majority of adult RGCs (Dupraz et al., 2013). In addition, the heterogeneity of the CNS neurons has been confirmed by SnRNA-seq, electrophysiological and morphological studies in the retina (Goetz et al., 2022). Based on this, we hypothesize that the RGCs analysed in this study belong to a regenerative neuronal subtype characterized by relatively rapid neurite outgrowth.

DRG neurons, which retain their regenerative capacity into adulthood, serve as a valuable model for studying axonal regeneration. However, different DRG neuron subpopulations exhibit varying regenerative abilities and express distinct molecular markers (Neumann and Woolf, 1999; Richardson and Issa, 1984). Similarly, at least 46 subtypes of retinal ganglion cells (RGCs) have been identified, each showing differential resilience to injury, with several genes implicated in subtype-specific injury responses (Tran et al., 2019). This cellular heterogeneity poses challenges for consistent and interpretable experimental outcomes. While RGCs represent a promising model for studying CNS regeneration, their diversity may introduce bias into the data, limiting the generalizability of findings (Sanes and Masland, 2015). In our current protocol, only about 3% of RGCs *in vitro*—those capable of extending long neurites—are amenable to analysis, suggesting that observed similarities between DRG neurons and RGCs likely pertain only to this regenerative RGC subpopulation. Notably, even under various *in vivo* regenerative treatments, only a small fraction of RGC axons regenerate over long distances (Petrova et al., 2020), raising the possibility that this same subset of intrinsically regenerative cells is being studied here.

### 4.2 Molecules implicated in the regeneration

We emphasize that the culture conditions used in this study did not include inhibitory or neurotrophic factors that could influence regeneration, apart from the laminin/PLL coating of the culture surface. However, we cannot entirely rule out the influence of cell type-specific requirements on regenerative capacity. Notably, even under identical culture conditions for RGC cultures only a subset of RGCs is capable of regenerating long axons, displaying microtubule density and polymerization kinetics comparable to those observed in peripheral DRG neurons. Furthermore, microtubule polymerization within these long neurites is predominantly anterograde. This is consistent with the established role of anterograde axonal transport as a key intracellular mechanism supporting axon elongation (Petrova and Eva, 2018).

Of the two components of anterograde transport, the slow component is responsible for delivering cytoplasmic proteins, enzymes, and actin at a rate comparable to that of axonal regeneration (Wujek and Lasek, 1983). Although both cell types examined in this study exhibited similar microtubule polymerization speeds, DRG neurons consistently regenerated longer neurites than RGCs within the same time frame, despite having comparable microtubule density and polymerization rates. This suggests that additional factors—such as the transport of vesicles and motor proteins—may play a critical role in neurite elongation, independent of polymerization dynamics. Local protein synthesis may also significantly influence regenerative capacity. For instance, the axon guidance molecule MAG promotes neurite outgrowth in DRG neurons from young animals and in RGCs, and this effect correlates with a developmental decline in endogenous neuronal cAMP levels. Elevating cAMP in older neurons has been shown to shift their growth state, enabling axonal extension even in the presence of myelin-associated inhibitors (Domeniconi and Filbin, 2005; Hannila and Filbin, 2008). Additionally, certain small molecules that act independently of cAMP have also been shown to promote neuronal protection and regeneration (Ma et al., 2010).

### 4.3 EB proteins

PNS neurons are capable of stabilizing their MTs, which facilitates the formation of regeneration-promoting structures. In contrast, CNS neurons lack this intrinsic regenerative ability and instead form growth-incompetent structures characterized by a disorganized MT network (Kulkarni et al., 2022). Fluorescently tagged EB proteins therefore serve as powerful tools for monitoring MT dynamics and, by extension, evaluating axonal regeneration. This approach is both efficient and non-toxic, allowing the visualization of MT density and orientation in living cells (Akhmanova and Hoogenraad, 2005; Galjart, 2010). In addition, investigating EB3 expression levels and its interactions with other molecules, such as fidgetin, could lead to the identification of novel therapeutic targets for promoting axonal regeneration (Ma et al., 2023). In this study, the EB3-GFP fusion protein enabled visualization of microtubule plus-end dynamics as distinct “comet-like” structures moving against a faint cytoplasmic background. Notably, increased EB3-GFP comet activity may reflect active remodeling of neurite segments (Kleele et al., 2014). Previous studies using EB3-GFP in cultured cells have shown that microtubules grow more slowly in neurons than in glial cells (Stepanova et al., 2003), and that EB3 movement is comparable across various neuronal compartments, including the soma, dendrites, axons, and growth cones. Consistent with these findings, we observed slower comet movement within the RGC soma. Regarding comet directionality, earlier studies in embryonic E17 mouse hippocampal neurons and E18 Purkinje neurons reported that approximately 65% of EB3 comets in proximal dendritic regions moved distally, while 35% moved retrogradely toward the soma. In more distal dendritic compartments and axons, however, most EB3 comets were oriented toward the growth cone. Similarly, our results showed that the majority of EB3 comets in regenerating neurites moved in the anterograde direction, suggesting that, at 4 days post-transfection (4 dpt), many neurites exhibit axon-like behavior (Baas et al., 1989). Conversely, retrograde polymerization observed in smaller neurites may indicate growth-phase dendrites, which could account for impaired elongation in some neurons due to mixed microtubule orientation.

Comet dynamics have been shown to be altered in two familial myotrophic lateral sclerosis models, with increased comet densities reported (Kleele et al., 2000). In unpolarized neurons such as those from the DRG, microtubule stabilization in a single neurite typically precedes axon specification. In cultured hippocampal neurons, the transition of a minor neurite into the future axon is marked by several key changes at the growth cone, including an increase in growth cone size, expansion of the peripheral lamellipodial veil, shortening of actin filaments (ribs), enhanced actin dynamics, and an increase in both the number and length of newly assembled dynamic microtubules. These microtubules subsequently penetrate both the central and peripheral regions of the growth cone (Bradke and Dotti, 1997; Conde and Caceres, 2009). Significantly, we observed clear microtubule penetration into the axons of both DRG and RGC neurons. However, parallel experiments using antibodies against classical axonal and dendritic markers—such as MAP2, MAP1B, tau, ankyrin, and neurofascin—failed to identify well-differentiated axons in cultured RGCs (unpublished data), despite the fact that these markers are readily detected in RGC axons *in vivo* (Van Wart et al., 2007). This discrepancy raises the possibility that RGC neurites grown *in vitro* may be unable to fully organize their cytoskeleton, potentially due to the absence of appropriate target-derived cues. Nevertheless, our findings suggest that the mechanisms underlying microtubule polymerization dynamics show a degree of similarity between the RGC subset we analyzed and DRG neurons. However, given the incomplete cytoskeletal organization of RGC neurites *in vitro* and the likelihood that key regulatory pathways depend on cell-type-specific signals or *in vivo* cues, these parallels should be interpreted cautiously. Additional mechanisms are expected to influence the extent and nature of regenerative responses in these different neuronal populations.

### 4.4 Mitochondrial movements

It is well established that mitochondria in healthy cells are dynamic organelles, and their movement is closely regulated by the energy demands of different cellular compartments. Deficiencies in mitochondrial transport have been implicated in a range of neurological disorders (MacAskill and Kittler, 2010), although the mechanisms underlying cytoskeleton-mediated transport remain incompletely understood. Several studies have suggested that the extent and directionality of mitochondrial movement are linked to neuronal regeneration capacity (Misgeld et al., 2007). Indeed, in neurodegenerative diseases, mitochondrial dysfunction can result in an insufficient ATP supply for microtubule motor proteins, disrupting mitochondrial axonal transport; enhancing microtubule-mediated trafficking may help prevent mitochondrial-induced damage (Esteves et al., 2014; Jang et al., 2024). In the present study, we demonstrate that MT polymerization occurs independently of mitochondrial transport. This opens the possibility for future investigations into how alterations in microtubule dynamics might influence mitochondrial behavior. Importantly, we show that it is feasible to simultaneously visualize both microtubules and mitochondria in adult CNS and PNS neurons derived from the same animal, offering a powerful platform for such studies.

In conclusion, this study provides new insights into the cytoskeletal mechanisms involved in axonal regeneration of adult CNS and PNS neurons under uniform experimental conditions. Future investigations could focus on other RGC subpopulations, particularly after neurite elongation or following regenerative treatments, to better understand subtype-specific responses. The methodology introduced here offers a novel and robust platform for studying adult RGC regeneration in vitro. Comparing them with DRG neurons from the same animals lays the groundwork for elucidating the role of axonal transport in regeneration. Notably, the observed variability in the regenerative capacity among RGCs supports the notion that these neurons exhibit heterogeneous responses to injury. This is consistent with findings in glaucoma, where some RGCs degenerate while neighboring cells survive.

## Data Availability

The original contributions presented in the study are included in the article/Supplementary material, further inquiries can be directed to the corresponding author.
